# From pond to platform: how *Synechocystis* sp. PCC 6803 became the default model cyanobacterium

**DOI:** 10.1128/jb.00535-25

**Published:** 2026-04-01

**Authors:** Sofia Doello, Jonas Hammerl, Karl Forchhammer, Martin Hagemann, Wolfgang R. Hess, Conrad W. Mullineaux, Annegret Wilde

**Affiliations:** 1 Interfaculty Institute of Microbiology and Infection Medicine, University of Tübingen 9188, Tübingen, Germany; 2 Molecular Genetics of Prokaryotes, Faculty of Biology, University of Freiburg 234634, Freiburg, Germany; 3 Spemann Graduate School of Biology and Medicine (SGBM), University of Freiburg 9174, Freiburg, Germany; 4 Department of Plant Physiology, University of Rostock 9187, Rostock, Germany; 5 Genetics and Experimental Bioinformatics, Faculty of Biology, University of Freiburg 98898, Freiburg, Germany; 6 School of Biological and Behavioural Sciences, Queen Mary University of London 4617, London, United Kingdom; Dartmouth College Geisel School of Medicine, Hanover, New Hampshire, USA

**Keywords:** *Synechocystis*, history, genetics, photosynthesis, carbon metabolism

## Abstract

Cyanobacteria are an ancient clade of phototrophic prokaryotes responsible for the initial oxygenation of the Earth’s atmosphere and still remain as major contributors to the productivity of the biosphere. Apart from their ecological importance, they are widely used as model organisms in photosynthesis research and show great potential for green biotechnology. Cyanobacteria are united by their specialized phototrophic metabolism but are otherwise extremely diverse in habitat, physiological adaptations, and morphology: they range from tiny unicellular species to complex multicellular forms with multiple specialized cell types. Out of all the vast range of cyanobacteria available, why did *Synechocystis* sp. PCC 6803 become the default laboratory model? Here, we recount its early history as a laboratory model and the key decisions that led to its establishment as the go-to cyanobacterium. We explain its advantages and limitations as a model organism, along with key advances in understanding that have been enabled by *Synechocystis* research. Finally, we discuss the role of *Synechocystis* in ongoing basic and applied research.

## INTRODUCTION AND EARLY HISTORY

In 1971, the Berkeley Culture Collection of “unicellular blue-green algae” consisted of more than 40 strains of cyanobacteria. *Synechocystis* sp. PCC 6803 (at this time named as *Aphanocapsa* N-1) was one of the 14 strains which were isolated at Berkeley from a local freshwater lake by R. Kunisawa in 1968 (1). This information can be found in nearly all documents on the history of *Synechocystis* sp. PCC 6803, but the life story of R. Kunisawa is not well known. Riyo Kunisawa was a Japanese-American microbiologist born in San Francisco who studied at the University of California, Berkeley. She came from a family of Japanese immigrants, and as a child, she was interned in a camp with her parents during World War II (https://80over80sf.org/80over80-stories/riyokunisawa). Despite her difficult early life, Riyo Kunisawa became a pioneer in cyanobacterial microbiology, and we gratefully acknowledge her contributions. In 1991, she retired, and five years later, in 1996, the strain she had isolated more than 25 years earlier became the first phototrophic organism to have its complete genome sequenced, by a group of Japanese researchers at the Kazusa DNA Research Institute in Chiba, Japan (2). This landmark achievement also made it the fourth genome of a cellular lifeform to be completely sequenced at that time (after *Haemophilus influenzae* [3], *Mycoplasma genitalium* [4], and *Mycoplasma pneumoniae* [5]).

Outgoing from the Berkeley strain collection, *Synechocystis* traveled around the world. It was deposited in the Pasteur Culture Collection (PCC) in 1968 with its current numbering (PCC 6803) and in the American Type Culture Collection (ATCC) as ATCC 27184, respectively. Sergey Shestakov and Galina Grigorieva from Moscow State University obtained several cyanobacterial strains from the Pasteur Culture Collection to test for genetic transformability, including *Synechocystis* sp. PCC 6803. The Shestakov laboratory demonstrated natural transformation for several cyanobacterial freshwater strains, including *Synechocystis* sp. PCC 6803 (6, 7). However, *Synechocystis* sp. PCC 6803 is particularly interesting because it can be grown on glucose in a fully heterotrophic mode; therefore, it is possible to inactivate photosynthetic genes. During the 4th International Symposium on Photosynthetic Prokaryotes in Bombannes, France, in 1982, John Williams (Wilmington, USA) and Sergey Shestakov discussed the idea of using *Synechocystis* sp. PCC 6803 as a model strain for studying photosystems (8). Subsequently, John Williams developed a method for targeted mutagenesis of photosynthetic genes in *Synechocystis* sp. PCC 6803 (9) using a glucose-tolerant (GT) non-motile strain, which was isolated from the strain ATCC 27184 and grew very well under photomixotrophic conditions. Although the PCC strain can also grow on glucose and several photosynthesis mutants have been created in this strain, the GT strain has become the dominant strain in photosynthesis research in many laboratories, primarily due to the pioneering work of Wim Vermaas in 1986 (10). Notably, it was the GT strain that was later sequenced by Kaneko et al. in 1996 (2). With advances in sequencing technology, several *Synechocystis* sp. PCC 6803 wild-type variants were re-sequenced and physiologically characterized (11–13), suggesting that growth of *Synechocystis* sp. PCC 6803 cultures in different laboratories worldwide have selected a significant number of wild-type variants with different properties, such as motility (13) and photosystem assembly (12). Differences in the phenotypes of different *Synechocystis* sp. PCC 6803 substrains, including a history of strain evolution, have been provided by Zavřel et al. (14). Due to the extensive body of literature on this model strain, we regret that it has not been possible to reference all significant contributions in this and the following sections. Any omissions are unintentional, and we sincerely acknowledge and appreciate the valuable work of researchers whose studies could not be cited here.

## UNIQUE FEATURES AND LIMITATIONS OF *SYNECHOCYSTIS* SP. PCC 6803

As discussed above, *Synechocystis* sp. PCC 6803 was first established as a model organism for photosynthesis studies because of its combination of genetic tractability and ability to grow photomixotrophically or heterotrophically on glucose. However, these features are not unique to *Synechocystis* sp. PCC 6803. For example, the marine species *Synechococcus* sp. PCC 7002 is also naturally transformable, can grow heterotrophically on glycerol, and has been used for a substantial body of photosynthesis-related work (15). In contrast, many other cyanobacterial model species lack the ability to grow heterotrophically. For example, *Synechococcus elongatus* PCC 7942 has become a classic model for systems such as the circadian clock (16); however, its inability to grow heterotrophically has greatly restricted its use in photosynthesis research. More generally, *Synechocystis* sp. PCC 6803 is metabolically versatile and can survive under a wide range of environmental conditions, making it an excellent model for studying cyanobacterial metabolism and stress responses. These points are discussed in detail in the sections below.

*Synechocystis* sp. PCC 6803 was the first cyanobacterium (and the first phototroph) to have its genome completely sequenced (2), securing its position as the default laboratory model for cyanobacteria. Although complete genome sequences are now available for hundreds of different cyanobacteria, the early establishment of *Synechocystis* sp. PCC 6803 as the default model means that more crucial background information is available for *Synechocystis* sp. PCC 6803 than for any other cyanobacterium. This remains a key reason for selecting *Synechocystis* sp. PCC 6803 as a model for molecular biological research in cyanobacteria, since so much information is readily available, for example, on the transcriptome, including transcriptional start sites and small regulatory RNAs (sRNAs) (17), the proteome (18–20), and the metabolome (21). Nevertheless, cyanobacteria are a large and diverse group of microbes, and other cyanobacteria have their own advantages for specific applications and as models for particular research fields. Such advantages include the particularly rapid growth of some species (22), the greater ecological significance of species including the marine *Synechococcus* and *Prochlorococcus* (23) and the freshwater *Microcystis aeruginosa* infamous for its toxin production (24), and the greater stability of protein complexes from the thermophilic *Thermosynechococcus elongatus*, which makes it the preferred model for structural biology (25). Some species exhibit physiological capabilities that are absent in *Synechocystis* sp. PCC 6803, including nitrogen fixation, multicellularity, and specialized cell differentiation (26).

## BIOLOGICAL AND TECHNOLOGICAL ADVANCES ENABLED BY *SYNECHOCYSTIS* SP. PCC 6803

### Insights into photosynthetic structure and function

**Fig 1:**
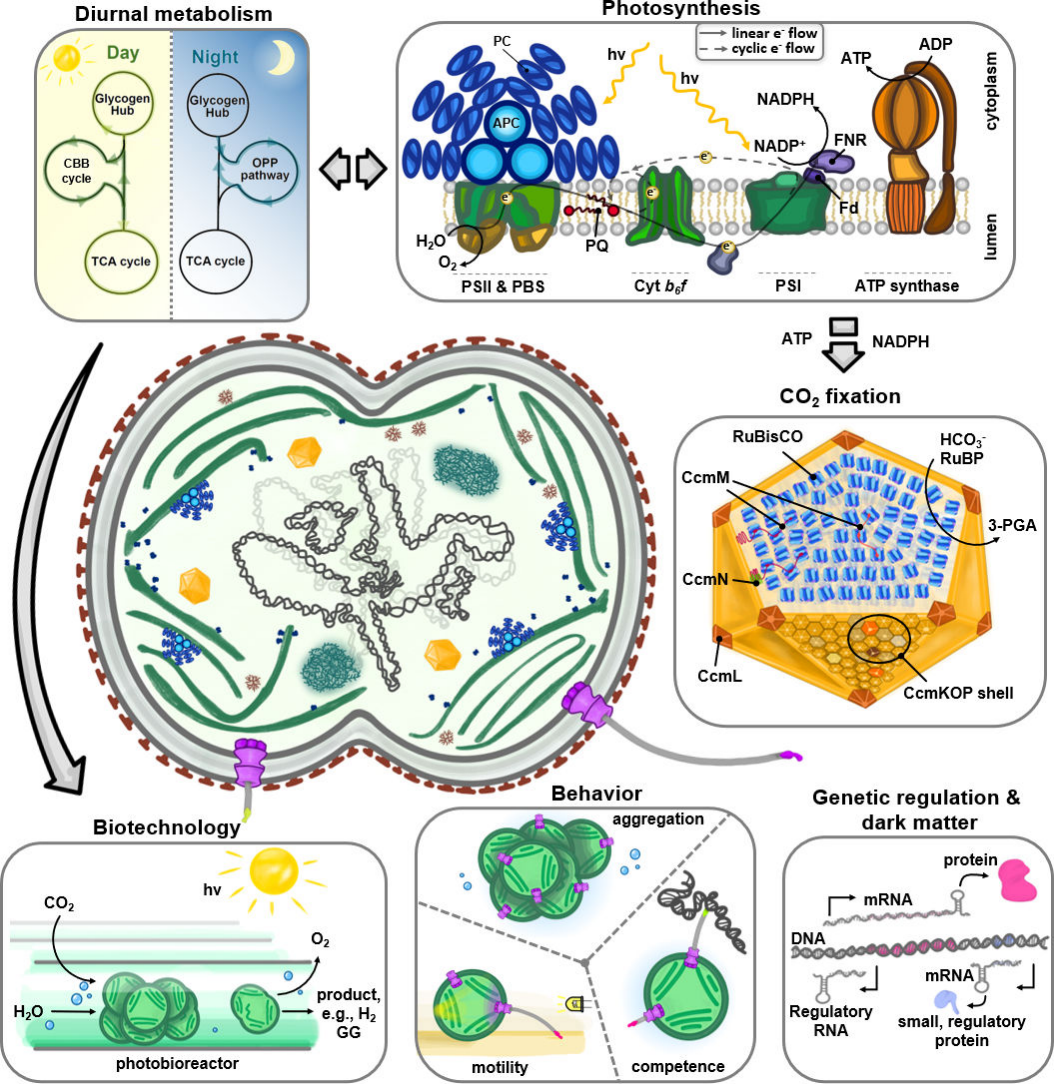
Overview of key research directions using *Synechocystis* sp. PCC 6803 as a model cyanobacterium. Summary of the main structural, physiological, genetic, and behavioral characteristics that have established *Synechocystis* sp. PCC 6803 as a leading model organism for cyanobacterial research. Core cellular functions include oxygenic photosynthesis, carbon, and nitrogen metabolism. Emerging research directions focus on primary metabolism and regulatory and adaptive processes, such as stress responses, circadian clock, behavior, and biotechnological applications.

Genetic accessibility and ease of production of photosynthetic mutants in *Synechocystis* sp. PCC 6803 established it as a major model organism in studies of photosynthetic function, despite the fact that *Thermosynechococcus elongatus* is often preferred for structural analysis. Numerous early studies used the methodology pioneered by Williams (9) to create photosynthetic null mutants that would have been lethal in many other oxygenic phototrophs (Fig. 1). These include mutants lacking functional photosystem II (10), later functional photosystem I (27), and both photosystems (28), enabled by the ability of GT *Synechocystis* sp. PCC 6803 to grow heterotrophically in Light-Activated Heterotrophic Growth (LAHG) conditions (27). Subsequently, Köbler et al. (29) described that at least one substrain of *Synechocystis* sp. PCC 6803 can grow in complete darkness. However, the genetic basis for this remains unclear. Photosynthesis mutants provided early insights into the roles of specific photosystem subunits, the plasticity of the cyanobacterial photosynthetic apparatus, and the assembly pathways of the photosystems. More recent highlights, again often based on the characterization of *Synechocystis* null mutants, include the identification of pathways for photosystem biogenesis and key proteins that enable them (30, 31), the identification of the FtsH1 protease (Slr0228) as a key factor in the photosystem II repair cycle (32), the identification of the orange carotenoid-binding protein as a photoprotective energy quenching factor (33), the characterization of complexes involved in delivering chlorophyll to nascent reaction centers (34), the characterization of the photoprotective roles of flavodiiron proteins (35), and the identification of many players in the cyanobacterial CO_2_-concentrating mechanism (36). Furthermore, mutagenesis approaches in *Synechocystis* sp. PCC 6803 have been widely used to characterize the complex set of alternative photosynthetic, respiratory, and hybrid electron transfer pathways in cyanobacteria (37–39). The advent of single-particle cryo-electron microscopy means that thermostability has become less critical for structural studies, leading to an increasing number of studies analyzing the structure of *Synechocystis* sp. PCC 6803 photosynthetic complexes (40, 41), and the ability to combine mutagenesis with high-resolution structural analysis promises further insights in the future. Although there are minor variations, the core photosynthetic complexes are strikingly conserved in all oxygenic phototrophs, meaning that insights from *Synechocystis* research are often applicable to plant chloroplasts, for example.

Beyond the structure and function of the photosynthetic apparatus, the photoautotrophic lifestyle of *Synechocystis* sp. PCC 6803 permeates every aspect of its physiology and metabolism and introduces distinctive differences from other bacterial model organisms. In the following sections, we consider *Synechocystis* sp. PCC 6803 as a model organism for cyanobacterial stress responses, behavior, and metabolism.

### *Synechocystis* sp. PCC 6803 as a model to study stress response among cyanobacteria

Adaptation to extreme environments and acclimation to fluctuating conditions among cyanobacteria are central research goals. Here again, the model *Synechocystis* sp. PCC 6803 has played a key role because of the availability of genetic tools (Fig. 1). Since *Synechocystis* sp. PCC 6803 was isolated from a lake in California, it is often regarded as a freshwater cyanobacterium. However, in fact, *Synechocystis* sp. PCC 6803 is a truly euryhaline strain that can grow at salinities up to twice that of seawater. It can accumulate the compatible solute glucosylglycerol (GG) (42). A search for salt-sensitive mutants led to the identification of the first genes for GG synthesis among cyanobacteria (43) and its turnover (44, 45). The primary functional characterization of GG synthesis genes permitted the definition of GG as a characteristic of truly marine strains and enabled the reconstruction of their phylogeny (46). During the last two decades, many omics technologies have been used to study the salt acclimation of *Synechocystis* sp. PCC 6803, leading to the identification of two-component systems involved in salt sensing (47, 48) and salt-regulated proteins, as well as the potential regulatory role of sRNAs (49).

The limited availability of ferrous iron (Fe^2+^) is considered a major limiting factor for cyanobacterial productivity in the open ocean (50). Many molecular aspects of the regulation of this important acclimation process have been resolved using *Synechocystis* sp. PCC 6803 as a model. These aspects include the identification of the transcription factor Fur as key to iron regulation and description of the entire Fur regulon (51); the identification of IsiA and the resolution of trimeric photosystem I and IsiA ring as the largest photosynthetic complex structure (52); the identification of IsrR as regulatory antisense RNA acting on the *isiA* mRNA (53) and of the sRNA IsaR1 targeting several different mRNAs (54); and the discovery of the major role of the protease FtsH3 as an epistatic master regulator of the iron stress response (55).

The stress responses toward many other metal ions have been analyzed with *Synechocystis* sp. PCC 6803 as a cyanobacterial model. Among them, the response to copper is highly important. In higher, non-physiological concentrations, it is toxic for cyanobacteria (recently reviewed in reference 56). However, trace amounts of copper are found in most of the media used to grow cyanobacteria because it is part of several redox systems in photosynthetic organisms. Of specific importance is the alternative, copper-dependent expression of the soluble electron carriers plastocyanin (PetE), which contains copper, and cytochrome *c*_6_ (PetJ), which contains iron, both of which can transfer electrons from the cytochrome *b*_6_*f* complex to photosystem I. Under copper-replete conditions, PetE is the preferred electron transporter, whereas under copper-deplete conditions, it switches to PetJ (57). The availability of copper is sensed in *Synechocystis* sp. PCC 6803 by the two-component system CopRS, with CopS as membrane-bound histidine kinase and CopR as soluble response regulator, which regulates the expression of genes for copper uptake and export, as well as other copper-containing proteins, including PetE (58, 59). Due to their tight control under different copper concentrations, the *petJ* and *petE* promoters (together with other metal-responsive promoters) have been frequently used to drive artificial gene expression in *Synechocystis* sp. PCC 6803 and other cyanobacteria for biotechnological purposes (reviewed in reference 60).

### Behavioral studies

Bacteria are highly prone to mutations because of their short doubling times. Consequently, laboratory strains can diverge phenotypically due to different laboratory-specific growth conditions. While such variation may remain inconspicuous when investigating conserved or essential cellular functions, it can become particularly significant in studies focusing on stress conditions or newly evolved or complex traits, such as behavioral responses. Already in the first description of *Synechocystis* sp. PCC 6803 by Stanier et al. in 1971 (1), the authors occasionally observed colony movement on plates. In addition, a highly cited cyanobacterial resource paper by Rippka et al. in 1979 (61) mentioned that one of the characteristics of *Synechocystis* sp. PCC 6803 is motility. More than 20 years later, Choi et al. analyzed *Synechocystis* sp. PCC 6803 motility in detail and demonstrated that this unicellular cyanobacterium senses the light direction rather than a change in light intensity, which is a hallmark of true phototactic response (62). In the early 2000s, pioneering work by two laboratories, led by Devaki Bhaya and Mashiko Ikeuchi, identified the fundamental role of type IV pili in *Synechocystis* sp. PCC 6803 motility. They were the first to elucidate the photoreceptors and chemotaxis-like genes involved in the regulation of motility (63–65). Further advancement revealed that spherical *Synechocystis* sp. PCC 6803 cells utilize their micro-optic properties to sense the direction of light (66), providing a physical basis for directional light perception at the single-cell level. Ongoing research has explored the complexity of signal transduction involving many different photoreceptors, structural proteins, enzymes, various regulators, and second messengers (67). While the second messengers cAMP and c-di-GMP clearly affect motility, they also affect other behavioral responses, such as aggregation (68), biofilm formation (69), and cell-cell communication (reviewed in reference 70).

One of the major challenges in studying behavioral responses is that different laboratories work with different substrains of *Synechocystis* sp. PCC 6803. In particular, strains originating from the GT wild type isolated by Williams (9) are non-motile and harbor mutations related to the function of type IV pili, whereas previously motile variants, such as the type strain from the Pasteur Culture Collection, can easily lose motility when propagated in different laboratories. One of the reasons for the selection of non-motile strains is that motile colonies appear blurry on plates, and people tend to select round colonies with clear boundaries. Furthermore, motile variants of *Synechocystis* sp. PCC 6803 stick together and form aggregates in liquid cultures and photobioreactors, making them unsuitable for biotechnological approaches and many physiological measurements. Thus, choosing the appropriate substrain for the respective research topic is fundamental when working with *Synechocystis* sp. PCC 6803, and most probably also with other (cyano)bacterial species. If provenance is lost, tests for motility and glucose tolerance are quick ways to establish which substrain is being used. Alternatively, PCR can be used to check for informative mutations, such as the 154 bp indel within and upstream of gene *slr2031*. This DNA segment is present in “PCC-M,” “PCC-P,” and “PCC-N” strains, but is lacking in GT-I and GT-S substrains (13). This 154 bp deletion was first noticed by Katoh et al. in 1995 (71).

### *Synechocystis* sp. PCC 6803 as a model for the study of cyanobacterial metabolism

Central metabolism in cyanobacteria encompasses the core biochemical pathways that sustain phototrophic and nocturnal metabolism (Fig. 1). During phototrophic growth, CO_2_ fixation and the assimilation of inorganic nutrients, especially nitrogen, drive the production of cellular building blocks. Catabolic pathways ensure cell survival in the absence of light or enable mixotrophic growth, in which organic nutrients from the environment are utilized. In this context, *Synechocystis* sp. PCC 6803 displays great metabolic versatility and is one of the few cyanobacteria capable of growth under strictly heterotrophic conditions. These methodological advantages have established *Synechocystis* sp. PCC 6803 as the model of choice for investigating cyanobacterial metabolism (72). The following sections highlight key aspects of metabolism for which *Synechocystis* sp. PCC 6803 has been used as a model system.

#### Central carbon metabolism and regulation

After the invention of oxygenic photosynthesis, the inorganic carbon-assimilating activity of cyanobacteria, algae, and plants decreased the available CO_2_ in the atmosphere to the present-day amount of only 0.04%. Cyanobacteria responded to the decline in CO_2_ by evolving a CO_2_-concentrating mechanism (CCM) to increase the CO_2_ in the vicinity of ribulose-1,5-bisphosphate carboxylase/oxygenase (RuBisCO). The main components of CCM were discovered using *Synechocystis* sp. PCC 6803 as a model, for example, the high- and low-affinity CO_2_-hydrating NDH1-like complexes (73) and the sodium-dependent bicarbonate transporter A, SbtA (74). Together with two other bicarbonate transporters, they increase the cellular concentration of bicarbonate by at least 100-fold. Bicarbonate then diffuses into the prokaryotic organelle carboxysome, where the carbonic anhydrase CcaA (75) releases CO_2_ near the encapsulated RuBisCO. The carboxysome shell is formed by different proteins, among which the structure of the hexameric CcmK1 was first elucidated in *Synechocystis* sp. PCC 6803 (76). In response to limiting CO_2_ availability, mainly the genes encoding the different bicarbonate and CO_2_ uptake systems are transcriptionally upregulated in *Synechocystis* sp. PCC 6803 (77), whereas in the model strain *Synechococcus elongatus,* the expression of carboxysome subunits and RuBisCO is also highly activated (78). It has been thought that through the CCM activity, the competing oxygenase function of RuBisCO is suppressed in cyanobacteria; however, using defined mutants, three routes to detoxify the toxic photorespiratory intermediate 2-phosphoglycolate (2-PG) and their essential roles have been identified in *Synechocystis* sp. PCC 6803 (79).

RubisCO and the associated Calvin-Benson-Bassham (CBB) cycle serve as a universal route for the assimilation of inorganic carbon in cyanobacteria (80). As mentioned above, external glucose can be utilized by *Synechocystis* sp. PCC 6803. Early ^14^C-labeling studies using the closely related strain, *Synechocystis* sp. PCC 6714, showed that glucose is mainly metabolized through the oxidative pentose phosphate (OPP) pathway (81). In addition, cyanobacteria have the enzymatic capability to perform the canonical glycolytic pathway, which provides organic carbon to the tricarboxylic acid (TCA) cycle. The TCA cycle in cyanobacteria is modified because the oxoglutarate dehydrogenase complex is absent. In *Synechocystis* sp. PCC 6803, alternative pathways can convert 2-oxoglutarate (2-OG) to succinate under certain conditions (82, 83). Consequently, the main function of the TCA cycle is related to the production of 2-OG, which serves as a precursor for nitrogen assimilation. It is believed that under light conditions, mostly the CBB cycle, photorespiration, lower glycolysis, and TCA cycle are active, while the OPP pathway mainly operates in the dark. However, recent flux analysis in *Synechocystis* sp. PCC 6803 showed that all anabolic and catabolic carbon routes can be active in the light, at least under dark/light transitions or under photoheterotrophic conditions (84).

Nevertheless, CCM activity and CO_2_ fixation are tightly regulated in response to fluctuating CO_2_ conditions. Many genes for the CCM, especially those encoding proteins of inorganic uptake systems, are activated under low carbon conditions using the transcriptional regulators NdhR (85), CmpR (86), RbcR (87), and SyCRP1 (88). These transcription factors sense CO_2_ availability via low molecular signals, whereby 2-PG and ribulose-1,5-bisphosphate signal low CO_2_, while 2-OG and cAMP are indicative of high CO_2_ (89, 90). In contrast to the CCM, carbon flux seems to be mainly regulated at the biochemical level because almost all enzymes involved in carbon metabolism show unchanged abundance under different CO_2_ conditions in *Synechocystis* sp. PCC 6803 (91). The PII-like protein, SbtB, has been identified in *Synechocystis* sp. PCC 6803 as a new regulator for carbon acclimation (90), which can directly interact with the SbtA transporter depending on whether AMP or cAMP is bound (92) and can also regulate glycogen accumulation in response to the second messenger c-di-AMP (93). Furthermore, SbtB contributes to the regulation of gene expression under variable CO_2_ conditions in *Synechocystis* sp. PCC 6803 (94). Furthermore, the activity of the CBB cycle is regulated by the CP12 protein under different redox conditions, such as light/dark changes, glucose additions, or different CO_2_ levels in *Synechocystis* sp. PCC 6803 (95–97). This strain expresses a canonical CP12, i.e., it harbors two conserved cysteine pairs that bind either glyceraldehyde-3-phosphate dehydrogenase 2 (Gap2) or phosphoribulokinase (PRK) under oxidizing conditions, whereas CP12 in *Synechococcus elongatus* PCC 7942 misses the PRK-binding cysteine pair. Hence, in the latter strain, the inactivation of PRK under oxidizing conditions is mainly due to internal redox cysteine pairs in the PRK structure (98) and to PRK binding of the central CP12 domain. Finally, the PirC protein has been identified in *Synechocystis* sp. PCC 6803 as a crucial regulator for the carbon flux from the CBB cycle into lower glycolysis, and it can inhibit phosphoglycerate mutase in the presence of high carbon-to-nitrogen (C/N) ratios due to a high 2-OG signal (99).

When photosynthetically fixed carbon exceeds the immediate anabolic demands of the cell, the surplus is channeled into the synthesis of storage compounds. Unlike most bacterial species, which typically synthesize only one type of carbon storage compound, *Synechocystis* sp. PCC 6803 can produce two chemically distinct reserves: glycogen and polyhydroxybutyrate (PHB). Among these, glycogen has been well established as a crucial energy and carbon store, particularly important during dark periods and nutrient starvation. In contrast, the physiological role of PHB in cyanobacteria remains unclear (100, 101). *Synechocystis* sp. PCC 6803 has served as a model to investigate the physiological role of glycogen and the regulatory mechanisms governing its biosynthesis. Mutants lacking glycogen have revealed that, beyond acting as reserve polymer, glycogen serves a buffering role in carbon metabolism and contributes to stress adaptation (102). Enzymatic studies have shed light on the regulation of glycogen biosynthesis in *Synechocystis* sp. PCC 6803. ADP-glucose pyrophosphorylase, which catalyzes the formation of ADP-glucose from glucose-1-phosphate, and glycogen synthases, which transfer glucose units to glycogen granules, are both subject to redox regulation (103). Recently, phosphoglucomutases, which interconvert glucose-1-phosphate and glucose-6-phosphate, have been identified as critical nodes connecting glycogen with central carbon metabolism, particularly during stress responses (104, 105).

#### Nitrogen assimilation and C/N balance

Cyanobacteria maintain a balanced cellular C/N ratio of approximately 5:1 during steady-state growth, necessitating the coordinated assimilation of nitrogen that parallels carbon fixation processes. As *Synechocystis* sp. PCC 6803 is incapable of atmospheric nitrogen (N₂) fixation, it relies entirely on the availability of combined nitrogen sources for growth. The primary nitrogen assimilation pathways and their regulatory mechanisms are highly conserved among cyanobacteria. The glutamine synthetase-glutamate synthase (GS-GOGAT) pathway predominantly mediates ammonium assimilation. Earlier pioneering studies on nitrogen metabolism in *Synechocystis* sp. PCC 6803 were initiated by the group of Francisco J. Florencio, focusing on the regulation of glutamine synthetase activity (106), whereas *Anabaena* and S*ynechococcus* strains were used during the late 20th century to elucidate the core aspects of nitrogen metabolism (107, 108). These investigations led to the identification of a global nitrogen control system regulated by the transcription factor NtcA, which was independently discovered in *Synechococcus* sp. PCC 7942 (109) and in *Nostoc* (previously *Anabaena*) PCC 7120 (110), with transcriptional co-activation by PipX, identified via yeast two-hybrid screening in *Synechococcus elongatus* PCC 7942 (111). However, it was in *Synechocystis* sp. PCC 6803 that the NtcA regulon was determined with high sensitivity, encompassing 51 activated and 28 repressed genes directly regulated by NtcA (112).

Concurrently with the characterization of NtcA, the PII signaling protein was defined as a key sensor of the cellular C/N status, with 2-OG serving as a signaling metabolite to report intracellular nutrient balance (113, 114). These insights have provided a comprehensive understanding of C/N metabolic control. At the transcriptional level, the regulatory interplay among PII, NtcA, and PipX constitutes a finely tuned network that integrates 2-OG signaling with cellular energy status (115, 116). Additional layers of regulation within nitrogen assimilation pathways have been identified, such as small regulatory RNAs (117). *Synechocystis* sp. PCC 6803 has also been successfully used as a model organism in metabolic flux analysis and genome-wide modeling of C/N metabolism (82, 118–120).

#### Metabolic dormancy and resuscitation

In non-diazotrophic cyanobacteria, the complete absence of combined nitrogen triggers a bleaching process known as chlorosis, in which cells enter a dormant state. This phenomenon, initially characterized in *Synechococcus elongatus* PCC 7942 (121), was later investigated in *Synechocystis* sp. PCC 6803, where the re-greening of chlorotic cells upon re-addition of combined nitrogen occurs in a synchronous, population-wide manner. Because of this combination of genetic tractability and physiological coherence, the molecular mechanisms behind the transitions into and out of metabolic dormancy could be studied in *Synechocystis* sp. PCC 6803 (122–124). A key cellular process for resuscitation of chlorotic cells is the tightly regulated utilization of glycogen reserves (125). The unique situation in which cells switch to heterotrophic metabolism even under illumination has allowed for the discovery of several regulatory mechanisms controlling the activity of glycogen catabolic enzymes, including phosphoglucomutases and glucose-6-P dehydrogenase (104, 126–128).

### Systems biology studies, CRISPR biology, and bioinformatic resources

Since *Synechocystis* sp. PCC 6803 was one of the first bacteria whose chromosomal sequence was determined (2, 129), along with the sequence of various plasmids (130–132), it became the primary model organism in several pioneering genome-wide studies among cyanobacteria (Fig. 1).

The need to make such data sets available in a suitable format is tightly linked to the development of appropriate databases. First and foremost, the establishment of CyanoBase (133) should be mentioned, although it is no longer maintained. A well-curated database to explore, analyze, and visualize genomic data and metabolic pathways is CyanoCyc (https://cyanocyc.org/) (134). To allow the comparative analysis of transcriptomic data sets, the CyanoEXpress database was developed (http://cyanoexpress.sysbiolab.eu/), providing access to curated genome-wide expression data (135, 136). Differential RNA-seq-type transcriptomic analyses (dRNA-seq) were used to generate detailed information about all promoters active at a given moment in *Synechocystis* sp. PCC 6803, driving the transcription of mRNAs and non-coding RNAs (17, 137). The term non-coding RNAs (ncRNAs) is used here to designate sRNAs, antisense RNAs (asRNAs), CRISPR RNAs (crRNAs), and all other RNA molecules that do not encode proteins. The dRNA-seq analyses established genome-wide maps of the 5,162 transcriptional start sites active in *Synechocystis* sp. PCC 6803 in at least one of 10 different growth conditions (available at http://cyanolab.de/software_downloads.html), leading to 4,091 transcriptional units on the chromosome and the plasmids pSYSA, pSYSG, pSYSM, and pSYSX. In this study, crRNAs were found to be among the most abundant transcripts in the cell, making *Synechocystis* sp. PCC 6803 is a model for CRISPR biology in cyanobacteria. *Synechocystis* sp. PCC 6803 possesses three different CRISPR-Cas systems, which are encoded together on plasmid pSYSA (138) and were classified as type I-D, type III-Bv, and type III-Dv systems. The co-occurrence of multiple CRISPR-Cas systems belonging to different biochemically distinct types can also be observed in many other cyanobacteria (139). The III-Dv CRISPR-Cas system is associated with cyclic oligoadenylate-mediated signaling (140), functions as a protein-assisted ribozyme (141), and is regulated by the redox-responsive transcription factor RpaB (142). This observation indicates that CRISPR-Cas systems, which are often regarded as relatively autonomous systems, can be more deeply integrated with the cellular machinery controlling gene expression (143, 144).

Information on the relevance of a specific gene under certain conditions is of high interest for fundamental and applied research. In a pioneering approach for cyanobacteria, CRISPR interference (CRISPRi) was established in *Synechocystis* sp. PCC 6803 targeting all 3,546 annotated protein-coding genes and 1,871 potential ncRNAs on the chromosome (145). In a subsequent study, the same group included plasmid-located genes and estimates of gene fitness under several different conditions (146) (available at https://m-jahn.shinyapps.io/ShinyLib/). It should be noticed that there is strong evidence for many, possibly hundreds, of additional genes in *Synechocystis* sp. PCC 6803 that were not previously modeled (147). In this regard, ribosome profiling analyses in connection with proteogenomic approaches are prolific in the precise identification of hitherto unidentified protein-coding regions in seemingly “empty” intergenic regions, within asRNAs, and even within annotated protein-coding genes, out of frame (148). These data can be assessed through an easily accessible, interactive web-based genome browser at https://www.bioinf.uni-freiburg.de/~ribobase/.

Other databases have been established to provide information on co-fractionating proteins or proteins and RNA molecules in density gradient ultracentrifugation experiments (149, 150) (available online https://sunshine.biologie.uni-freiburg.de/GradSeqExplorer/ and https://synecho-rapdor.biologie.uni-freiburg.de).

Based on a systematic analysis of genomic, biochemical, and physiological characteristics, metabolic modeling approaches using flux balance analysis could be developed. These tools allow the prediction of essential metabolic pathways and reactions and the identification of inconsistencies in the existing annotation (151). In continuation of this work, they demonstrated that genome-scale metabolic models of *Synechocystis* sp. PCC 6803, together with appropriate constraints, are suitable for describing and predicting the quantitative aspects of phototrophic growth (152).

### Biofuel and bioproduct synthesis as a chassis organism

In recent years, cyanobacterial research has received a boost due to the introduction of these organisms as green cell factories, that is, as production strains that can potentially be used for the sustainable production of biofuels and feedstock using atmospheric CO_2_ and sunlight. *Synechocystis* sp. PCC 6803 has emerged as a pivotal model organism for the development of phototrophic production strains that convert atmospheric CO₂ and light into biofuels, bulk chemicals, and high-value biopolymers. Its extensively characterized physiology, fully sequenced genome, and the availability of sophisticated genetic tools, a wide range of suitable antibiotics, and marker recycling strategies—together with a broad and experienced research community—make *Synechocystis* sp. PCC 6803 particularly suitable for systematic metabolic engineering and proof-of-concept studies. Engineered *Synechocystis* 6803 strains have been constructed to synthesize a range of fuel molecules and platform chemicals directly from CO₂, including ethanol (153, 154), isobutyraldehyde and isobutanol (155), fatty acids (156), sucrose (157), hydroxypropionate (158), and isoprene (159, 160), demonstrating the versatility of this cyanobacterium as a phototrophic cell factory. In parallel, native storage and osmoprotective compounds that accumulate to high levels in *Synechocystis* sp. PCC 6803 can be further enhanced by targeted pathway and regulatory engineering, enabling increased formation of compatible solutes such as sucrose and GG (161), the nitrogen-rich polymer cyanophycin (162), and PHB (163), serving as a starting point for bioplastic development.

Beyond fuels, simple platform chemicals, and polymers, *Synechocystis* sp. PCC 6803 particularly has also been used in the realm of terpenoids to produce complex diterpenoids such as the forskolin precursor 13R-manoyl oxide, as well as cyanogenic glucosides like dhurrin, demonstrating that the host can support multi-step heterologous pathways for pharmaceutically relevant metabolites (164, 165). Several studies have further established *Synechocystis* sp. PCC 6803 as a platform for sesqui- and monoterpenes, including bisabolene, valencene, patchoulol, limonene, and related sesquiterpenoids, which are of interest as flavors, fragrances, and potential high-energy biofuel candidates (166–168).

In addition to these engineered products, *Synechocystis* sp. PCC 6803 has been exploited for the tailored biosynthesis of carotenoids and xanthophylls, for example, lutein and violaxanthin, by introducing plant carotenoid biosynthetic genes, thereby enabling the light-driven production and functional characterization of nutraceutically and cosmetically relevant pigments (169).

These examples illustrate the potential of *Synechocystis* sp. PCC 6803 is not only for the sustainable production of energy carriers but also for the generation of biobased materials, specialty chemicals, and polymer precursors in a single phototrophic chassis, even though current productivities in many cases remain below the threshold required for economic feasibility and thus primarily serve to guide the design of future, more robust cyanobacterial production platforms (170).

## CURRENT AND FUTURE QUESTIONS

For prokaryotes, cyanobacteria have an unusually complicated cell architecture. *Synechocystis* sp. PCC 6803 remains at the center of efforts to understand cyanobacterial cell organization and the factors that control it. The application of cellular electron tomography (171) and cryo-electron tomography (172) has given a major boost to the understanding of *Synechocystis* sp. PCC 6803 cell architecture. *Synechocystis* sp. PCC 6803 null mutants continue to inform on the drivers of cyanobacterial cell architecture, exemplified by the demonstration that the CurT protein has major influences on thylakoid membrane conformation (173), cell division, and the partitioning of the thylakoid system (174). Fluorescence microscopy combined with specific labeling of crucial cell components is well-established in *Synechocystis* (173, 175), although it is more challenging than in many other organisms due to the background fluorescence from the photosynthetic pigments. Major open questions address the problem of sorting proteins to the different membrane systems (176) and the pathways of lipid trafficking and membrane remodeling (177). Due to its genetic tractability and status as the default cyanobacterial research model, we expect *Synechocystis* sp. PCC 6803 to remain key to understanding cyanobacterial cell organization. Another prolific field for discoveries is the elucidation of the functions of the many previously unidentified small proteins (147, 148). This has been exemplified recently by establishing the roles of CP12, SbtC, NblD, or AtpΘ in the regulation of nitrogen, carbon, and energy metabolism (96, 97, 178–180). Further open research questions concern the mechanisms of post-translational gene regulation and RNA metabolism. The control of gene expression through transcription factors and two-component systems has been relatively well explored in cyanobacteria, as in many other bacteria. However, there is a large discrepancy between studies on enterobacteria and Gram-positive models, such as *Bacillus subtilis* and *Staphylococcus aureus*, regarding post-transcriptional regulation. The existence and importance of such regulation involving sRNAs and asRNAs have been demonstrated in cyanobacteria, mainly in *Synechocystis* sp. PCC 6803, and relevant work has been performed to identify the involved proteins. Therefore, the preconditions have been established now to unravel the post-transcriptional regulatory networks for a cyanobacterial cell.

*Synechocystis* sp. PCC 6803 has served as an excellent model and has laid the foundation for a molecular understanding of the cyanobacterial cell. This has opened a new gateway to understanding the key representatives of this group of organisms, such as nitrogen fixers, symbionts, marine plankton, and toxin producers. What makes cyanobacteria so robust in natural environments, and why do they continue to dominate vast regions of the Earth after existing for more than three billion years? Re-isolating strains from their natural environments could provide valuable insights into the extent to which laboratory adaptation has led to the loss or modification of traits present in wild populations. This approach has been successfully applied to, for example, *Synechococcus elongatus* PCC 7942 (181), highlighting its potential to uncover functions that are no longer present in domesticated laboratory strains. Once we have answers to these questions and gain a comprehensive and global understanding of cyanobacteria, the prerequisites will be in place for an even more effective application as green cell factories.
